# Neonatal Cholestasis – Differential Diagnoses, Current Diagnostic Procedures, and Treatment

**DOI:** 10.3389/fped.2015.00043

**Published:** 2015-06-17

**Authors:** Thomas Götze, Holger Blessing, Christian Grillhösl, Patrick Gerner, André Hoerning

**Affiliations:** ^1^Department for Pediatric and Adolescent Medicine, Friedrich-Alexander University of Erlangen-Nuremberg, Erlangen, Germany; ^2^Department for Pediatric and Adolescent Medicine, Albert-Ludwigs-University Freiburg, Freiburg, Germany

**Keywords:** neonatal cholestasis, neonatal jaundice, conjugated hyperbilirubinemia, biliary atresia, kasai procedure

## Abstract

Cholestatic jaundice in early infancy is a complex diagnostic problem. Misdiagnosis of cholestasis as physiologic jaundice delays the identification of severe liver diseases. In the majority of infants, prolonged physiologic jaundice represent benign cases of breast milk jaundice, but few among them are masked and caused by neonatal cholestasis (NC) that requires a prompt diagnosis and treatment. Therefore, a prolonged neonatal jaundice, longer than 2 weeks after birth, must always be investigated because an early diagnosis is essential for appropriate management. To rapidly identify the cases with cholestatic jaundice, the conjugated bilirubin needs to be determined in any infant presenting with prolonged jaundice at 14 days of age with or without depigmented stool. Once NC is confirmed, a systematic approach is the key to reliably achieve the diagnosis in order to promptly initiate the specific, and in many cases, life-saving therapy. This strategy is most important to promptly identify and treat infants with biliary atresia, the most common cause of NC, as this requires a hepatoportoenterostomy as soon as possible. Here, we provide a detailed work-up approach including initial treatment recommendations and a clinically oriented overview of possible differential diagnoses in order to facilitate the early recognition and a timely diagnosis of cholestasis. This approach warrants a broad spectrum of diagnostic procedures and investigations including new methods that are described in this review.

## Introduction

Neonatal physiological jaundice is a common and mostly benign symptom. It typically resolves 2 weeks after birth. Neonatal cholestasis (NC), however, indicated by a conjugated hyperbilirubinemia, is never benign and indicates the presence of a severe underlying condition. A defect of the intrahepatic production or the transmembrane transport of bile, or a mechanical obstruction preventing bile flow leads to an accumulation of bile components in the liver, in the blood and extrahepatic tissues. The incidence of NC is ~1 in 2500 live births (1). Of the various conditions that can present with NC, biliary atresia (BA) represents the major cause and has been reported to occur in 35–41% of the cases followed by progressive familial intrahepatic cholestasis (PFIC) (10%), preterm birth (10%), metabolic and endocrinological disorders (9–17%), Alagille syndrome (AS) (2–6%), infectious diseases (1–9%), mitochondriopathy (2%), biliary sludge (2%), and, finally, idiopathic cases (13–30%) (2, 3). The rapid diagnosis of BA is paticularly important because early surgical intervention by hepatoportoenterostomy before 2 months of age correlates with better long-term outcome (4–8). Unfortunately, the diagnosis of NC is often delayed and the average age at diagnosis of BA is about 60 days in USA and Germany (9, 10). For these reasons, the fractionated bilirubin must be determined in any infant presenting with prolonged jaundice lasting longer for 14 days of age for term and 21 days for preterm infants with or without depigmented stool. Once conjugated hyperbilirubinemia is identified, a systematic approach is the key to reliably achieve a rapid diagnosis, so that the specific and often life-saving therapy can be promptly initiated. This review offers not only a systematic diagnostic approach but also highlights new diagnostic techniques that have decreased the proportion of idiopathic cases in favor of previously under-reported conditions, such as PFIC, bile acid synthesis defects, and mitochondrial disorders (2). The most frequent causes of NC are discussed and rare congenital infectious or neonatal disorders are summarized in an adjacent table.

### A diagnostic approach to NC and recommendations for initial management

**Figure 1:**
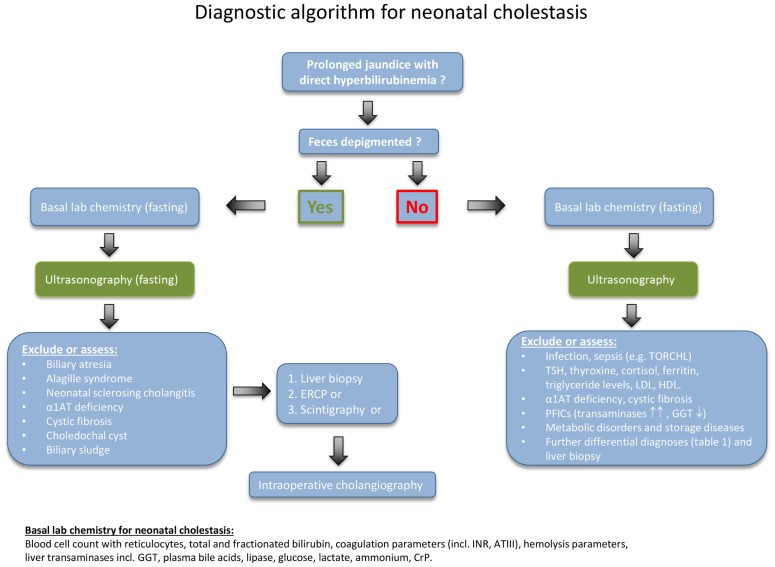
**Diagnostic algorithm for neonatal cholestasis**. After confirming direct hyperbilirubinemia, biliary atresia as the most frequent disorder must rapidly be excluded. Hence, the abdominal ultrasound examination (fasting) is of central importance for the evaluation of a cholestatic infant and should be obtained as early as possible. Liver structure, size, dilated bile ducts, gall bladder (size, wall thickness, triangular cord sign), identification of extrahepatic obstructive lesions (e.g., choledochal cyst, gallstones, sludge), ascites, spleen size, situs abnormalities, and vascular malformations should be determined by an experienced operator. If feces is not depigmented and the ultrasound examination remains inconclusive, the diagnostic procedure needs to be quickly expanded as indicated in the text box on the lower right.

In cases of prolonged neonatal jaundice, the fractionated bilirubin needs to be determined as a first step (Figure 1). A conjugated bilirubin of more than 1 mg/dL in combination with a total bilirubin of <5.0 mg/dL, or a conjugated bilirubin fraction of >20% of the total, if total bilirubin is >5.0 mg/dL, indicates NC. A parenteral report of depigmented feces suggests an extrahepatic obstructive process. For clarification, the stool should be seen by a physician in any case.

First of all, conditions or complications that require immediate treatment should be detected. Therefore, a series of basic blood tests need to be performed (as listed at the bottom of Figure 1). Further important initial steps are a fasting ultrasonography, a liver biopsy, and a hepatobiliary scintigraphy. If these tests are inconclusive, then the next step is to undertake an intraoperative cholangiogram or ERCP. In some cases, the liver biopsy may need to be repeated 3–4 months after birth, because several of the diseases follow a dynamic course (11, 12). In patients with advanced disease, insufficient hepatic synthetic function and hypovitaminosis, a vitamin K-dependent bleeding disorder may occur. Therefore, an oral supplementation with vitamin K (1 mg/day), vitamin A (1500 U/kg/day), vitamin D (cholecalciferol; 500 U/kg/day), and vitamin E (50 U/kg/day) should be initiated immediately.

### Disorders causing neonatal cholestasis

#### Obstructive Bile Duct Disorders

Biliary atresia is the most frequent and severe cause of NC. In a few cases, it may be part of a syndrome and associated with other congenital malformations such as polysplenia (100%), situs inversus (50%), or cardiac anomalies (50%) and/or vascular malformations, e.g., preduodenal portal vein (60%) (13).

Biliary atresia is an ascending inflammatory process of the biliary tree leading to progressive obliterative scarring of the extrahepatic and intrahepatic bile ducts, resulting in biliary cirrhosis (14). Only early surgical treatment can stall biliary cirrhosis, which is why rapid identification of BA is crucial.

The exact etiopathogenesis of BA is still unknown and is suggested to involve environmental, infectious, and genetic factors (15). In murine models, pre- or perinatal infections with rotavirus (16, 17), cytomegalovirus (15), and reovirus (18) were shown to infect and damage bile duct epithelia giving support to the hypothesis of a primary cholangiotropic viral infection as the initiating event of BA. In humans, however, studies focusing on the detection of viral infections at the time of diagnosis have produced conflicting results (19). In addition, alloimmune events mediated by liver infiltrating maternal effector T lymphocytes (microchimerism) have also been proposed to play a part in the pathogenesis of BA (20).

Infants with BA present with a conjugated hyperbilirubinemia, elevated liver transaminases including GGT and depigmented or pale stool. An abdominal ultrasound should be performed early to exclude other extrahepatic obstructive lesions such as choledochal cysts, gallstones, or bilary cast. The finding of a small or even absent gallbladder is suggestive of BA. In the majority of the cases, the presence of a structure ≤15 mm in length located in the expected region showing irregular margins without a well-defined wall excludes a normal gallbladder (21). A pre- and postprandial ultrasonography facilitates the demonstration of a physiologic gall bladder contraction. A triangular periportal echogenic structure (>3 mm in thickness) in the liver hilum is called the “triangular-cord-sign,” a finding associated with high sensitivity (73–100%) and specificity (98–100%) of BA (22, 23). In addition, assessing the hepatic arterial subcapsular flow by color Doppler ultrasonography has recently been reported to enable the discrimination of BA from other causes of NC (24).

The diagnostic gold standard of BA is a percutaneous liver biopsy followed by an intraoperative cholangiogram if inconclusive (11). Liver biopsy leads to the diagnosis in 79–98% of cases (25). Histology is characterized by inflammatory cell infiltrates around bile ducts, portal tract fibrosis, accumulation of bile typically presenting as bile plugs, and bile duct proliferation. ERCP may serve as a reliable and safe additional diagnostic tool (10, 26, 27) exhibiting a sensitivity of up to 86–92% and a specificity of 73–94% (10, 28). Hepatobiliary sequence scintigraphy is associated with a high sensitivity (83–100%) but lacks specificity in many cases (33–80%), limiting its usefulness to discriminate between BA and other non-surgical conditions (29). Additional non-invasive and other quite promising studies focus on the identification of serum markers that may reliably distinguish BA from other forms of NC (30, 31). Song et al. (30) found that the expression of the apolipoproteins Apo C-II and Apo C-III to be upregulated in the serum of infants with BA, while Zahm et al. (31) analyzed elevated patterns of circulating micro-RNAs and identified the miR-200b/429 cluster as a potential biomarker.

Treatment of BA consists of a hepatoportoenterostomy to enable biliary drainage (32). The success rate of this procedure is closely associated with the age of the infant at time of surgery (4, 7–9). Most reliable predictors for successful surgery and long-term outcome with the native liver are the normalization of conjugated bilirubin and AST 2 months after surgery (33). Although the problem of late diagnosis has been defined for some years, surgery within the first 2 months of age has still not been universally achieved in many centers/countries (9, 10, 34). In Taiwan, a stool color card system led to a decline of late referral (35), which is one reason why it is regarded as a simple and promising screening approach to early the identification of BA (36).

*Alagille syndrome* accounts for 2–6% of infants with NC (2, 3). Liver histology typically demonstrates a paucity of interlobular biliary ducts. Bile ductular proliferation as observed in BA is absent. Mutations in the JAG-1 gene are responsible for more than 90% of cases of AS; others have mutations in the gene encoding for the NOTCH-2 receptor (37, 38). The main diagnostic criteria of AS are typical facial signs such as a broad forehead, deep set eyes, and a pointed chin giving the face a triangular appearance (77–98%), an ocular embryotoxon (61–88%), cardiac abnormalities (85–97%), and butterfly vertebrae (39–87%). Minor criteria are growth (50–87%) and/or developmental delay (16–52%), renal disease such as renal cysts, renal artery stenosis, and tubular acidosis (40–73% cases), and exocrine pancreatic insufficiency (40%) (39, 40). Early development of hepatocellular carcinoma (HCC) may occur regardless of the presence of cirrhosis. Thus, patients with AS should be screened for HCC with alpha-fetoprotein and ultrasonography every 6 months.

Cystic, ectatic alterations of the intra- and/or extrahepatic bile duct system represent a further cause of NC.*Choledochal cysts* account for 2–3% of infants with NC and are surgically treatable (2). According to Todani, five anatomic variants have been described, but most infants show dilatation of the common bile duct (Todani type 1, 50–80% of biliary cysts) (41). *Caroli’s disease* is associated with multifocal, segmental, and saccular or fusiform dilatations of the medium and large intrahepatic bile ducts without affecting the common bile duct (28). These malformations may be limited to one liver lobe, most commonly the left one. Involvement of both the medium intrahepatic bile ducts (Caroli’s disease) and the small interlobular bile ducts results in the more frequent *Caroli syndrome*. Caroli syndrome is associated with ciliopathies and frequently presents with congenital hepatic fibrosis. This condition may also include renal abnormalities, e.g., autosomal recessive polycystic kidney disease (ARPKD), juvenile nephronophtisis, Joubert syndrome, and others (42). The diagnosis is established by ultrasound, ERCP, and/or MRCP (28). Administration of ursodeoxycholic acid (UDCA) decreases bile stasis and prevents the formation of intrahepatic cholelithiasis. Liver transplantation is a curative therapy (43).

*Neonatal onset of sclerosing cholangitis* is characterized at ERCP by a varying degree of stenotic and focally dilated intrahepatic bile ducts with rarification of segmental branches (44, 45). Liver transplantation is the only treatment option. In a few cases, it is associated with ichthyosis, and hence termed as neonatal ichthyosis and sclerosing cholangitis (NISCH) syndrome. NISCH is caused by mutations of the CLDN1 gene encoding for Claudin-1, a tight-junction protein. The Claudin-1 defect leads to an increased paracellular permeability and hepatocellular damage caused by toxicity of paracellular bile regurgitation (46).

*Idiopathic neonatal giant cell hepatitis* is a differential diagnosis after exclusion of all other causes of NC (47). Histology findings are non-specific, and include the formation of syncytial multinucleated hepatic giant cells, variable inflammation, with infiltration of lymphocytes, neutrophils, and eosinophils and lobular cholestasis – changes that are also seen in other conditions, such as α1ATD or PFIC (47).

The non-cholestatic entities *Dubin-Johnson syndrome* (defect in the MRP2 gene) (48) and *Rotor syndrome* (defect in the OATP1B1/OATP1B3 gene) (49) need to be distinguished from NC. These conditions also manifest with direct hyperbilirubinemia but the excretion of serum bile acid is unimpaired. In Dubin–Johnson syndrome, biliary excretion of conjugated bilirubin via the MRP-2 channel is impaired, whereas in Rotor syndrome, there is a defect in the hepatic storage of conjugated bilirubin, which then leaks into the plasma. Diagnosis and differentiation between these conditions are achieved by urinary coproporphyrin analysis (50, 51).

### Alterations in the hepatocellular bile acid transport system

The PFIC conditions are a heterogeneous group of autosomal recessive disorders characterized by mutations in hepatocellular bile acid transport-system genes leading to impaired bile formation. About 10% of infants that present with NC suffer from PFIC-1 and -2 (2). Three types of PFIC have been identified: PFIC1 and PFIC2 usually appear in the first months of life, whereas onset of PFIC3 typically arises later in childhood. Infants with PFIC1 or PFIC2 present with NC, therapy resistant pruritus and coagulopathy due to vitamin K malabsorption. A characteristic feature of these conditions is that the serum gamma-glutamyltransferase activity is normal or even low in PFIC1 and PFIC2 patients, but elevated in PFIC3 patients. PFIC1 is caused by impaired bile salt secretion due to defects in the ATP8B1 gene that encodes the FIC1 protein – a protein that stabilizes the hepatocellular membrane by transfer of aminophospholipids (52). Mutations in the FIC1 gene also cause benign recurrent intrahepatic cholestasis (BRIC1, Summerskill syndrome), which is characterized by recurrent episodes of cholestasis that may not necessarily lead to liver cirrhosis (53). Mutations in the ABCB11 gene encoding the bile salt export pump (BSEP) protein lead to PFIC2 (52, 54). Defects in ABCB4 that encodes the multidrug resistance 3 protein (MDR3) impair biliary phospholipid secretion and result in PFIC3 (52, 55). Diagnosis is based on immunostaining of BSEP (PFIC2) and MDR3 (PFIC3) in liver biopsies as well as the analysis of biliary lipid composition and genotyping for PFI mutations causing PFIC1. UDCA helps to postpone biliary cirrhosis in the PFIC conditions. An early partial external biliary diversion normalizes serum bile acids and relieves pruritus for children with PFIC1 and 2 (56). This procedure decelerates disease progression until liver transplantation is needed.

### Metabolic disorders and storage diseases

Neonatal cholestasis is caused by a number of *metabolic disorders* with *cystic fibrosis (CF)* and *alpha-1-antitrypsin deficiency (α1ATD)* being the most common. Although infants with CF are more likely to present with meconium ileus or steatorrhea with failure to thrive, 5% of patients with CF manifest with NC (57). α1ATD is inherited in an autosomal recessive trait and, similarly to CF, estimated to affect 1 in 2500–5000 children (58). The proportion of infants with α1ATD in cases of NC is about 1–17% (2, 3). A pathologic polymerization of AAT within the endoplasmatic reticulum results in an intrahepatocellular accumulation of the misfolded α1AT molecule leading to progredient liver fibrosis/cirrhosis. Hence, serum α1AT concentration is typically decreased. However, a normal α1AT level does not exclude the diagnosis because α1AT is an acute phase protein and may be raised by inflammatory processes. Therefore, the deficient α1AT-variant needs to be identified by protease inhibitor (PI) typing using polyacrylamide isoelectric focusing (59). While liver involvement in childhood is mainly associated with the PI-ZZ genotype (60), the PI-MZ and PI-SZ types are both characterized by respiratory symptoms (e.g., recurrent pneumothoraces) (61). The pathogenesis of the pulmonary manifestation differs from liver disease. In the lung, severe deficiency of the elastase inhibitor AAT results in an uninhibited proteolytic activity of elastase predisposing to a chronic obstructive disease with development of a panacinar emphysema. However, within the first two decades of life, liver dysfunction represents the major threat whereas pulmonary disease in not a major concern (62). Liver transplantation is curative and the treatment of choice for α1ATD.

*Inherited disorders of bile acid synthesis* cause ~2% of persistent cholestasis in infants (63). Cholestasis is thought to result from an imbalance and inadequate production of primary bile acids. This results in an accumulation of aberrant hepatotoxic bile acids and intermediary metabolites leading to an impaired bile flow (64). In contrast to other causes of NC, the serum bile acid levels are normal. Enzyme defects result in a specific pattern of metabolites that can be assessed by analysis of urinary cholanoids (bile acids and bile alcohols) (65, 66). Molecular–genetic analysis of the genes encoding the bile acid-CoA amino acid *N*-acyltransferase (BAAT) and bile acid-CoA ligase (SLC27A5) is possible (67). Peroral treatment with primary bile acids (cholic acid, not UDCA) leads to normalization of liver function in most patients (68).

*Storage disorders* and disorders of *lipid metabolism*, including Gaucher disease and Niemann–Pick type C disease (NPD), contribute to ~1% of infants with NC (2). In Gaucher disease, deficiency of the lysosomal β-glucocerebrosidase results in the impaired recycling of cellular glycolipids. The consequence is an excessive storage of glucocerebroside in the liver, lung, spleen, bone, and bone marrow (69). Infants develop anemia, thrombocytopenia, hepatosplenomegaly, and, in some cases, bone infarcts and aseptic bone necrosis. Therapeutic options for type I Gaucher disease are an enzyme-replacement therapy or a substrate reduction therapy with oral Miglustat that inhibits the glucosylceramide synthase (70). The diagnostic procedures for the confirmation of Gaucher disease and NPD are summarized in Table 1.

**Table 1:** **Summary of the differential diagnoses and diagnostic approaches**.

Disease	Diagnostic approach	Genetic analysis
**BILE DUCT OBSTRUCTION**		
Structural
Biliary atresia	US, liver biopsy, ERCP, hepatobiliary scintigraphy, intraoperative cholangiogram	Wildhaber (71), Mieli-Vergani and Vergani (4)
Alagille syndrome	Typical facial features, chest x-ray (butterfly vertebrae), ophthalmology, echo, liver biopsy, cholesterol ↑	*JAG1, NOTCH2* genes; Vajro et al. (39), Turnpenny and Ellard (40), Kamath et al. (38)
Choledochal cyst	US, ERCP, MRCP	Todani et al. (41)
Caroli’s disease/syndrome	US (liver and kidneys), ERCP, MRCP if >1 year of age, PKHD1 gene (ARPKD)	Adeva et al. (72), Harring et al. (43), Krall et al. (73)
Gallstones or biliary sludge	US, ERCP	
Neonatal sclerosing cholangitis	ERCP, liver biopsy	Baker et al. (44), Girard et al. (45)
Hepatocellular
Idiopathic neonatal giant cell hepatitis (NGCH)	Histological diagnosis after exclusion of other causes	Torbenson (47)
Progressive familial intrahepatic cholestasis (PFIC)	Liver biopsy, genetic analysis	*ABCB4, ABCB11, ATP8B1*; Jacquemin (52), Kubitz et al. (54)
	GGT (↓– → in types 1 + 2, ↑ in type 3)	
**METABOLIC DISORDERS, STORAGE DISEASES, AND OTHERS**		
Cystic fibrosis	Newborn screening (not in Germany), trypsinogen content in stool, genetic analysis	*CFTR* gene; Sokol and Durie (57)
A1AT deficiency	A1AT levels ↓	*SERPINA1* gene analysis for prenatal diagnosis; Perlmutter (59), Fregonese and Stolk (58)
	PI analysis (type ZZ, SZ, MZ)	
Inborn errors of bile acid synthesis	Urinary bile acid analysis, molecular–genetic analysis	Clayton et al. (66); *BAAT and SLC27A5* gene, Setchell et al. (67)
Gaucher disease	AP ↑, β-glucocerebrosidase ↓, chitotriosidase ↑, BM biopsy: “crinkled paper” cytoplasm and glycolipid-laden macrophages, foam cells (Gaucher cells)	Rosenbloom et al. (69)
Niemann–Pick type C	Filipin positive reaction (detection of cholesterol in fibroblasts), genetic testing, chitotriosidase ↑	*NPC1, NPC2* gene; Patterson et al.(74)
Wolman disease, LAL deficiency	Lysosomal lipase acid ↓ ↓ in PBMC	*LIPA* gene; Zhang and Porto (75)
Mitochondrial disorders	Fasting and postprandial lactate, plasma lactate/pyruvate ratio >20, functional assays, genetics	*SCO1, SUCLG1, BCS1L, POLG1, C10ORF2, DGUOK*, and *MPV17* gene mutations; Fellman and Kotarsky (76), Wong et al. (77)
Neonatal Intrahepatic cholestasis caused by citrin deficiency (NICCD)	Citrulline ↑, *α*-fetoprotein ↑, and ferritin ↑	*SLC25A13* gene; Lu et al. (78), Kimura et al. (79), Song et al. (80)
Peroxismal disorders (Zellweger’s spectrum and others)	Zellweger’s: Typical craniofacial dysmorphism, mental retardation, hepatomegaly, glomerulocystic kidney disease, cataracts, pigmentary retinopathy	Moser et al. (81, 82)
	VLCFA ↑, pattern of plasmalogenes, phytanic acid, pristanic acid	
Tyrosinemia	Newborn screening, urinary excretion of succinylacetone ↑, 4-hydroxy-phenylketones, and δ-aminolevulinic acid ↑ Cave: HCC (AFP ↑) (83)	*FAH* gene, de Laet et al. (84)
Classic galactosemia	Newborn screening, galactose-1-phosphate uridyl transferase activity in red blood cells ↓ ↓	Mayatepek et al. (85)
Congenital disorders of glycosylation (CDG)	Dysmorphic facies, convergent strabism, inverted mammils, mental retardation, seizures, dystrophy, hepatomegaly, hepatic fibrosis/steatosis, cyclic vomiting and diarrhea, coagulopathy, protein losing enteropathy with hypoalbuminemia (CDG1b)	Jaeken (86), Freeze (87, 88), Linssen et al. (89)
	Lab chemistry: Triglycerides ↑, ATIII ↓, factor XI ↓, protein C and S ↓, Transferrin IEF	
**ENDOCRINE DISORDERS**		
Hypothyroidism	Newborn screening (TSH ↑)	Hanna et al. (90)
Panhypopituitarism	Glucose ↓, Cortisol ↓, TSH ↓, fT4 ↓, IGF1 ↓, IGFBP ↓	Binder et al. (91), Karnsakul et al. (92)
**TOXIC OR SECONDARY DISORDERS**		
Parenteral nutrition-associated cholestasis (PNAC), drugs (e.g., anticonvulsants)	Exclusion of other causes	Hsieh et al. (93)
**IMMUNOLOGICAL DISORDERS**		
Gestational alloimmune liver disease (GALD)	Ferritin ↑ ↑ (>1000 μg/L), buccal mucosal biopsy and liver biopsy (iron deposition?), MRI (extrahepatic iron deposition?)	Rand et al. (94)
Neonatal lupus erythematosus	Transplacental passage of ANA, anti-RoSSA, anti-La/SSB, anti-U1RNP antibodies	Hon and Leung (95)
	Echo, ECG (congenital heart block)	
Haemophagocytic lymphohistiocytosis (HLH)	Fever (>7 days), hepatosplenomegaly with liver dysfunction, pancytopenia, sCD25 (>2400μg/mL), ferritin (>500 μg/L), triglycerides (>3 mmol/L), hypofibrinogenemia (<150 mg/dL), serum cytokine levels of both IFNg (>75 pg/ml) + IL-10 (>60 pg/ml) ↑	Lehmberg and Ehl (96), Xu et al. (97)
**INFECTIOUS DISORDERS**		
Sepsis, urinary tract infections, TORCH, hepatitis A–E, EBV, HIV, Echo, adeno, coxsackie virus, Parvo B19, HHV6-8, VZV, syphilis, leptospirosis	PCR, microbiology, serology, ophthalmologic examination (toxoplasmosis, CMV, rubella)	Kosters and Karpen (98), Bellomo-Brandao et al. (99), Robino et al. (100)
**VASCULAR MALFORMATIONS**		
Portosystemic shunts	US, MRI, LE ↑, unexplained galactosemia, hyperammonemia, manganemia	Bernard et al. (101)
Multiple hemangioma	US, MRI	Avagyan et al. (102), Horii et al. (103)
Congestive heart failure	Echo (heart anomalies, e.g., in Down syndrome), US, liver histology	Arnell and Fischler (104)
**MISCELLANEOUS**		
Genetic disorders	Trisomy 21, Trisomy 18	
ARC syndrome	Arthrogryposis multiplex congenita, facial dysmorphia, dystrophia, renal tubular acidosis, cholestasis, platelet dysfunction, ichthyosis	*VPS33B* gene; Smith et al. (105), Eastham et al. (106)
Aagenes syndrome	Lymphedema cholestasis syndrome 1 (LCS1)	Drivdal et al. (107)
Microvillus inclusion disease (MVID)	Life threatening congenital watery diarrhea of secretory type. Histology: microvillus atrophy, detection of PAS+ granules and CD10+ lining in LM and inclusion bodies in EM	*MYO5B* gene, Ruemmele et al. (108)
Neonatal leukemia	AML ≫ ALL	Van der Linden et al. (109)

*AFP, alpha-fetoprotein; ALP, alkaline phosphatase; BM, bone marrow; DD, differential diagnosis; ERCP, endoscopic retrograde cholangiopancreaticography; EM, electronmicroscopy; HCC, hepatocellular carcinoma; MRI, magnetic resonance imaging; LE, liver enzymes; LM, light microscopy; PBMC, peripheral blood mononuclear cell; US, ultrasound; VLCFA, very long chain fatty acids*.

*Wolman disease* and *cholesterol ester storage disease (CESD)* are rare autosomal recessive disorders with an estimated incidence of 1 in 40,000 live births (75). Their identification is especially important as an enzyme-replacement therapy with human recombinant lysosomal acid lipase (LAL) is now available and improves the natural course, thus offering a possibility for long-term survival (78, 79). *Wolman disease* may manifest as NC, and clinically appears within the first 2–4 months of age with hepatosplenomegaly, liver fibrosis, adrenal calcification and insufficiency, malabsorption, diarrhea, and poor weight gain as common features. Wolman disease usually takes a fatal course, with death within 12 months of age. Both diseases result from a deficiency of the LAL that usually catalyzes the degradation of cholesteryl esters and triglycerides that are delivered to the liposomes by a LDL-receptor mediated endocytosis. LAL-deficiency thus leads to elevated serum triglycerides and LDL, an intralysosomal accumulation of cholesteryl esters, triglycerides, and other lipids in various tissues, predominantly in macrophages. Whereas a loss of function mutation in the LIPA gene causes Wolman disease, CESD results when residual activity of the LAL enzyme is retained. Symptoms of *CESD* may be non-specific in early infancy. It is felt that the incidence of CESD is currently underestimated as it easily can be misdiagnosed as non-alcoholic fatty liver disease (NAFLD) later in childhood (75). Both entities show microvesicular steatosis on liver histology. Identification of Maltese cross-type birefringence (stored liquid crystals of cholesteryl esters) on frozen sections, and/or the detection of the lysosomal-associated membrane proteins 1 and 2 (LAMP) around the lipid droplets suggest CESD (110). Diagnosis is based on LIPA gene sequencing and assessment of LAL levels in PBMCs (111).

*Mitochondrial disorders* manifesting early in life often have a hepatic involvement, and in the majority of cases more than one organ such as the muscle or the nervous system is affected (76). A recent study reported that ~2% of infants with NC suffer from a mitochondrial hepatopathy (2). Clinical manifestations and the degree of organ involvement varies. This condition often remains undetected until exacerbation is precipitated, typically following an intercurrent viral infection. Histological findings include glycogen deprivation of the hepatocytes, microvesicular lipid accumulation, and signs of fibrosis/cirrhosis. Among the mitochondriopathies, Pearson syndrome is most often presents with liver manifestations (76). The diagnostic work-up should include genetic testing as well as functional assessment of the respiratory chain enzyme complexes in both liver and muscle biopsies (77). Of note, liver injury caused by any NC can secondarily affect OXPHOS activity. As a consequence, a compromised mtDNA complex activity may, therefore, not necessarily serve as proof for a mitochondriopathy related liver disease. Further rare metabolic causes are summarized in Table 1.

## Conclusion

A variety of disorders can present with cholestasis during the neonatal period. In all cases of neonatal jaundice lasting longer than 14 days, the measurement of fractionated bilirubin must be performed. BA is the most common entity leading to NC. It must be differentiated from other causes of cholestasis promptly because early surgical intervention before 2 months of age results in a better patient outcome. Previously underdiagnosed disorders comprise the PFICs, hepatic mitochondriopathies, and also, presumably, the LAL deficiency that causes Wolman disease and CESD. Early identification of the possible disorders is important, and prompt referral to a pediatric hepatology unit is essential.

## Conflict of Interest Statement

The authors declare that the research was conducted in the absence of any commercial or financial relationships that could be construed as a potential conflict of interest. No external funding was secured for this study.

## Abbreviations

AFP, alpha-fetoprotein; ALL, acute lymphoblastic leukemia; ALP, alkaline phosphatase; ALT, alanine aminotransferase; AML, acute myeloid leukemia; ANA, anti-nuclear antibodies; ARPKD, autosomal recessive polycystic kidney disease; AST, aspartate transaminase; ATIII, antithrombin III; α1AT, α1-antitrypsin; BA, biliary atresia; BM, bone marrow; BSEP, bile salt export pump; CDG, congenital disorders of glycosylation; CESD, cholesterol ester storage disease; CF, cystic fibrosis; CMV, cytomegalovirus; CrP, C-reactive protein; ECG, electrocardiogram; ERCP, endoscopic retrograde cholangiopancreatography; GALD, gestational alloimmune liver disease; GGT, gamma-glutamyltransferase; HCC, hepatocellular carcinoma; HIV, human immunodeficiency virus; HDL, high-density lipoprotein; HLH, hemophagocytic lymphohistiocytosis; HSV, herpes simplex virus; IEF, isoelectrofocusing; IGF, insuline-like growth factor; IGFBP, insuline-like growth factor binding protein; IVIG, intravenous immunoglobulin; LCS1, lymphedema cholestasis syndrome1; LDH, lactate dehydrogenase; LDL, low-density lipoprotein; LIPA, lysosomal lipase acid; MCP, medium chain triglycerides; MRCP, magnetic resonance cholangiopancreatography imaging; MRI, magnetic resonance imaging; NAFLD, non-alcoholic fatty liver disease; NGCH, idiopathic neonatal giant cell hepatitis; NICCD, citrin deficiency; NPD, Niemann–Pick disease; OXSPHOS, respiratory chain oxidative phosphorylation; PBMC, peripheral blood mononuclear cells; PCR, polymerase chain reaction; PFIC, progressive familiary intrahepatic cholestasis; PNAC, parenteral nutrition-associated cholestasis; PNALD, parenteral nutrition-associated liver disease; PTT, partial thromboplastin time; SMA, smooth muscle antibody; TNP, total parenteral nutrition; TSH, thyroid-stimulating hormone; US, ultrasonography; VLCFA, very long chain fatty acids.
